# Revascularization with BYCROSS atherectomy device- protocol of a prospective multicenter observational study

**DOI:** 10.1186/s42155-023-00404-8

**Published:** 2023-12-05

**Authors:** Dominik Liebetrau, Joerg Teßarek, Florian Elger, Sebastian Zerwes, Viktoria Peters, Christian Scheurig-Münkler, Alexander Hyhlik-Dürr

**Affiliations:** 1 Vascular Surgery, Medical Faculty, University of Augsburg, Stenglinstrasse 2, 86156 Augsburg, Germany; 2 Vascular Surgery, Bonifatius Hospital Lingen, Wilhelmstraße 13, 49808 Lingen (Ems), Germany; 3 Thoracic and Vascular Surgery, Medical Faculty, University Medical Center Goettingen, Robert-Koch-Straße, 4037075 Goettingen, Germany; 4 Department of Diagnostic and Interventional Radiology, University Hospital of Augsburg, Augsburg, Germany

**Keywords:** Critical limb ischemia, Endovascular revascularization, Mechanical thrombectomy, Thromboaspiration, Limb salvage, Crossing the occlusion device, Bi-directional rotational atherectomy, BYCROSS™ atherectomy device

## Abstract

**Background:**

The BYCROSS™ device is a novel device intended for use in atherectomy of the peripheral arterial disease (PAD). With the BYCROSS™ atherectomy system, also prolonged calcifying lesions can be treated in a minimally invasive manner, which was previously reserved for bypass surgery. The aim of this study is to collect additional clinical data on safety and performance of the BYCROSS™ from patients undergoing revascularization of severely stenotic or occluded peripheral arterial vessels with the BYCROSS™.

**Methods and design:**

This is an investigator-initiated national prospective multicenter observational study in patients with PAD. Sixty patients (20 per center) with PAD with stenosis higher than 80% or complete occlusion (de novo or recurrent stenosis) of vessels below the aortic bifurcation (min 3 mm vessel diameter) will be recruited. Three vascular surgery centers are participating in the study. The primary efficacy endpoint is procedural success, defined as passage of the occlusion through the BYCROSS device, and safety outcomes, explicated as freedom from device-related serious adverse events (SADEs). Secondary endpoints include primary and secondary patency rates, change in Rutherford classification, and freedom from amputation at 3 and 12 months.

**Discussion:**

The BYCROSS atherectomy system may be a novel device for the minimally invasive treatment of prolonged calcified lesions previously reserved for bypass surgery. This national prospective multicenter observational study could represent another step in demonstrating the efficancy and safety of this device for treatment of PAD.

**Trial registration:**

#DRKS00029947 (who.int).

**Protocol approval id:**

#22–0047(Ethics Committee at Ludwig-Maximilians-University Munich).

## Background

Approximately 230 million people are affected by vascular occlusive disease [1] worldwide. Overall, the incidence of PAD has steadily increased in recent years. As a result, the proportion of symptomatic patients requiring treatment is also increasing [2]. The focus has been on endovascular therapy as "first-line" therapy [3]. The BEST-CLI Trial could change the perspective. The results showed bypass surgery (even if an adequate great saphenous vein (GSV) is present) could provide better outcomes compared with endovascular treatment. The incidence for death or major adverse limb event (MALE) was significant lower in the bypass group with adequate GSV [4]. In contrast to the BEST-CLI trial, the BASIL-2 trial showed that endovascular treatment was associated with better outcomes (reduction in MALE) compared with bypass (vein). At the BEST-CLI Trial newer endovascular techniques, such as atherectomy, were not specifically mentioned and included in the comparison.

In addition to classical balloon angioplasty [5, 6], atherectomy is a procedure for the treatment of complex and persistent lesions [7, 8]. This method has been used for a variety of occlusive procedures [9, 10]. Despite the widespread use of these therapies, there is still considerable room for improvement as each available atherectomy system has its own limitations. (for example, in very long calcified lesions, complete occlusions, or the diameter of the created canal) [11]. In addition, atherectomy is associated with certain risks such as distal embolism, vascular injuries as well as perforation.

**Fig. 1:**
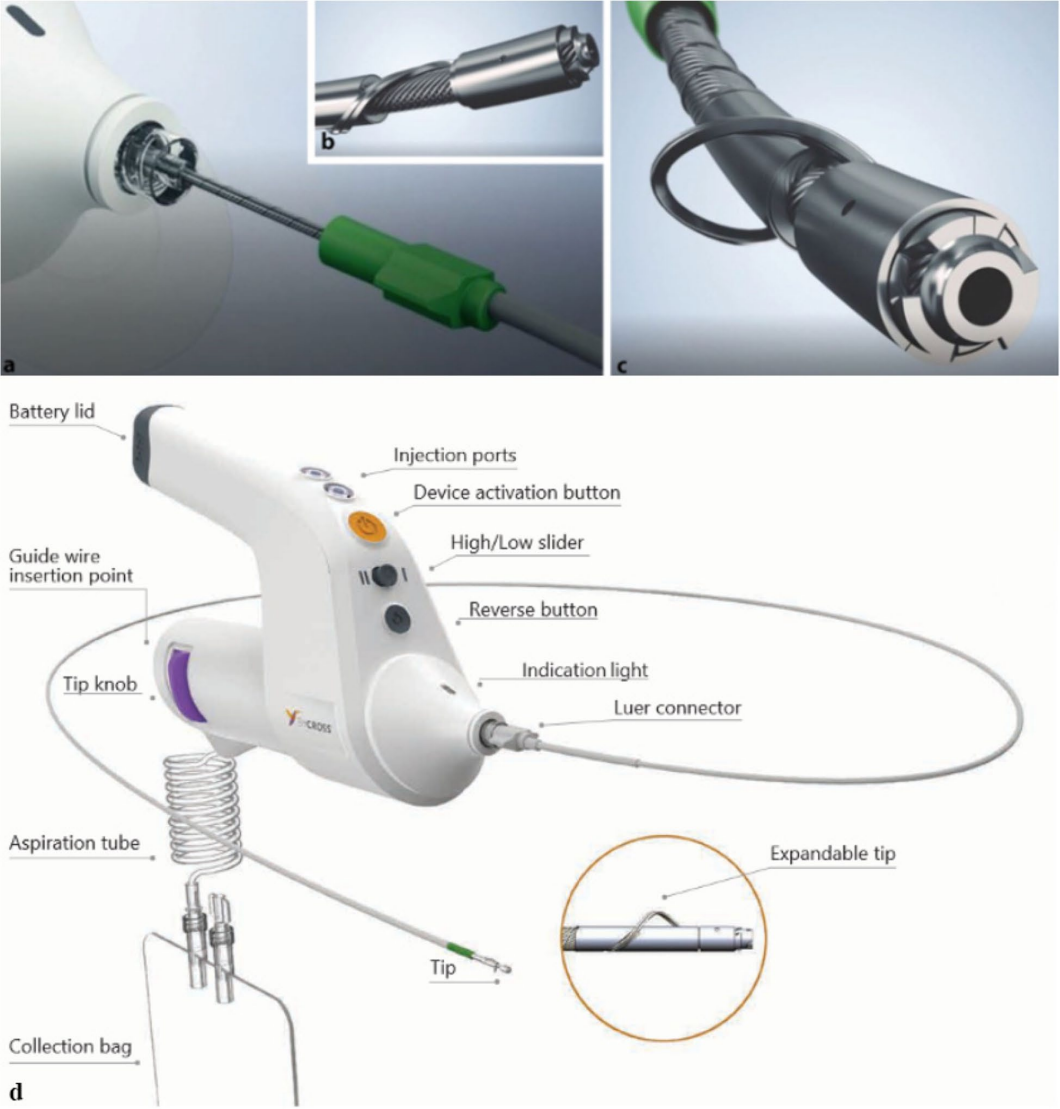
Special features of the ByCross® system: The system is advanced into the target vessel in a 6-French sheath (**a**) attached to the handle, through which the suction pump housed in the handle simultaneously with the porous shaft below the tip (**b**), which follows the principle of the Archimedean screw, aspirates and transports debris. The catheter tip has a variable diameter. This measures between 1.9 mm (**b**) and 4.7 mm (**c**) with the nitinol cutting wire extended. **d** Overview of Bycross atherectomy device, (Courtesy of Taryag Medical Inc., Israel

The innovative BYCROSS™ atherectomy system (TARYAG-MEDICAL GmbH, 14 Ha'Ilan st Or-Akiva, 3,065,101, Israel; Fig. 1) used in this study, represents a further development of existing atherectomy systems. During development, several limitations encountered in other systems were identified and eliminated.

## Methods

### Study aim

The aim of this prospective national multicenter observational study is to collect further clinical data on the safety and performance of the BYCROSS™ atherectomy system and to monitor treatment success after one year.

In the future, promising catheter-based treatments using the Bycross System could be made available to more patients. This could eliminate many more invasive procedures (e.g., bypass surgery), thereby increasing patient safety.

### Ethics

The study is being conducted in accordance with the Declaration of Helsinki on Research Involving Human Subjects and in compliance with the ICH Principles of Good Clinical Practice and additional local guidelines. This study was reviewed and approved by the Ethics Committee at Ludwig-Maximilians-University Munich, project-nr: 22–0047.

### Study design

**Fig. 2:**
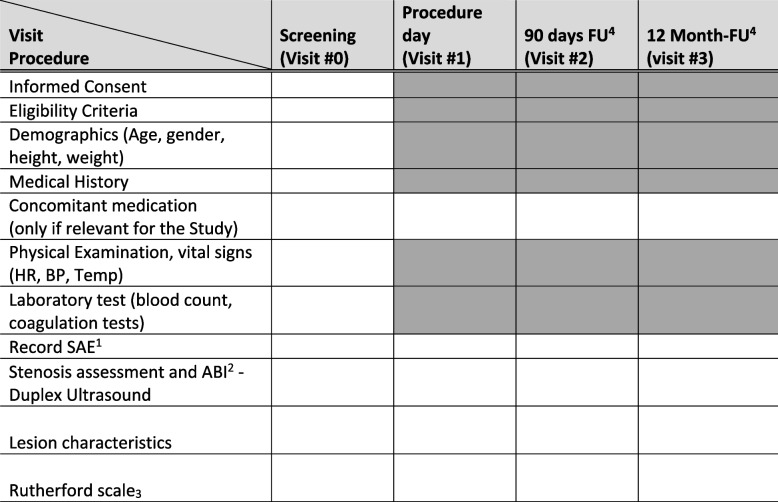
Study Activity Flow Chart. ^1^*SAE* Serious adverse event, ^2^*ABI* Ankle-Brachial-Index, ^3^Rutherford scale [12]; ^4^*FU* Follow-up

The study is designed as a multicenter (3 centers), prospective, single arm, observational study. The success of the procedure (primary endpoint) will be assessed in each participating subject, as well as safety outcomes (defined as freedom from serious adverse events associated with the device (SADE)). A total of 60 (target 20 each center) subjects with a symptomatic PAD will be enrolled in the study. After written informed consent, subjects will be continuously enrolled (registered). Subjects will be followed for a period of 12 months, including follow-up after, and follow-up 90 days after the procedure. The detailed study procedures are shown in the flowchart of study activities (Fig. 2).

### Inclusion and Exclusion criteria

Table 1 shows the inclusion and exclusion criteria.

**Table 1:** Overview of Inclusion and exclusion criteria

Inclusion criteria
a. Age ≥ 18
b. Subject has documented symptomatic chronic peripheral vascular disease at a vessel below the aortic bifurcation
c. Severely stenotic occlusion target vessel (stenosis ≥ 80%)
d. Subject has been informed on the nature of the study and has provided informed consent
e. Subject is capable of meeting study requirements including presences at follow-up visits
Exclusion criteria
a. Vessels of the cardiopulmonary, coronary or cerebral circulations
b. Undersized vessel diameters (< 3 mm)
c. Perforation of the vessel distally or proximally to the occlusion segment prior atherectomy
d. Subintimal position of the guiding catheter or the guidewire
e. Use in stents or stent grafts if the guidewire has become threaded at any point in the wire mesh of stent or stent graft or the lining of the stent graft
f. Target is at vessel segment which includes tortuous course with radius of curvature < = 40 mm
g. Access pathway includes tortuous course with radius of curvature < = 25 mm, in specific extremely sharp aortic bifurcation
h. In aneurysmatically altered iliac vessel segments
i. If the introducer sheath, the guide catheter, the guidewire or the BYCROSS™ sustains any visible damage, especially kinking
j. In the fracture areas of broken stents
k. Known or suspected allergy to any of the components of the system or to a medicinal product to be administered in connection with the planned procedure
l. Persistent vasospasm
m. Patient is pregnant

#### Study procedure

**Fig. 3:**
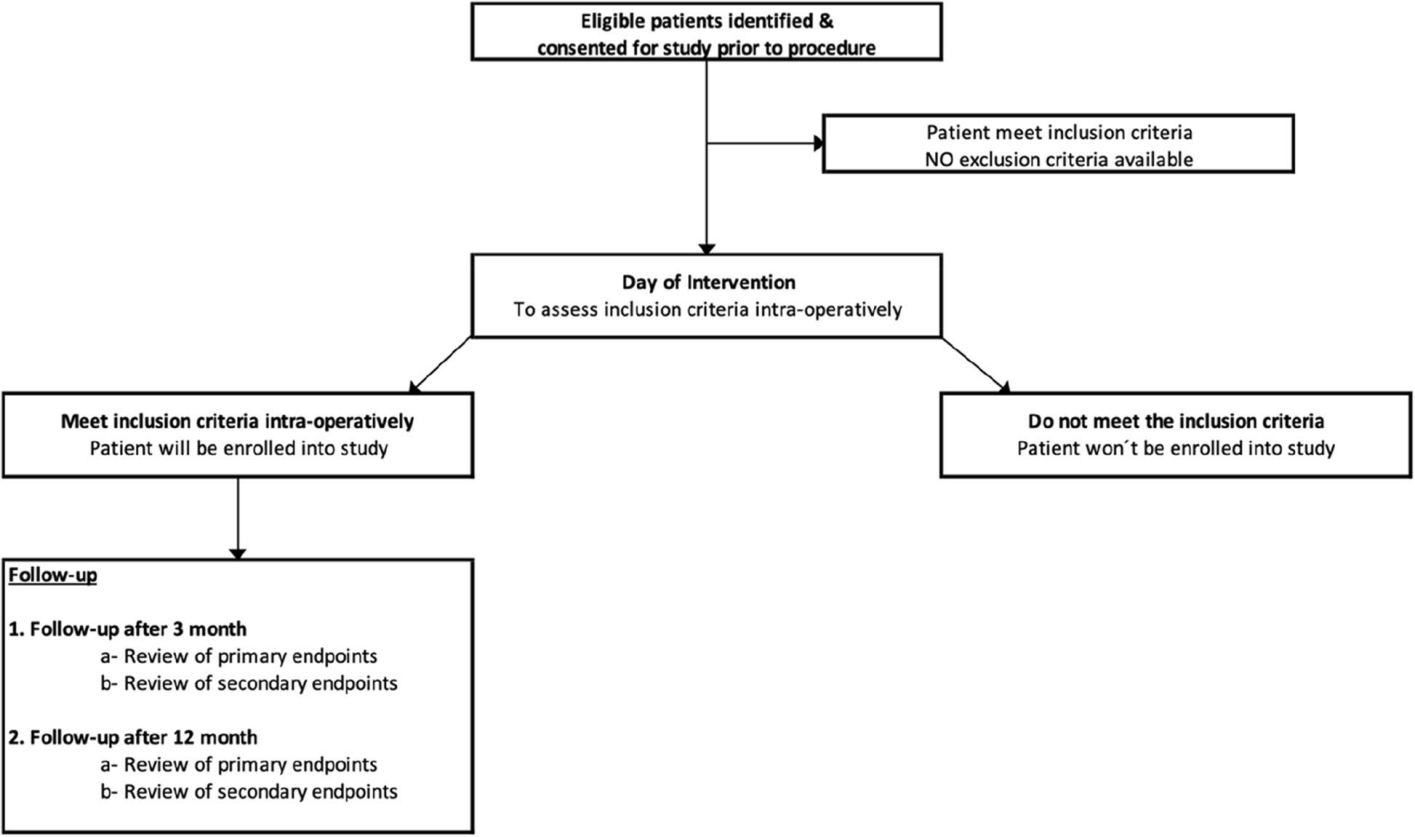
Participant flow through study

Schedule of events for this study are shown in Fig. 3. The study duration for each subject will be approximately 12 months, as follows:

1. Screening of individuals who meet the inclusion criteria
2. Procedure with BYCROSS™ atherectomy system
3. Follow-up at 90 days after procedure
4. Follow-up at 12 months after-procedure

### Endpoints

The primary performance endpoint is technical success: Passage of the occlusion through the BYCROSS® device and residual stenosis after atherectomy of ≤ 50% compared to the reference diameter to allow angioplasty and/or stenting if needed, and complete procedural success with residual stenosis of ≤ 30%. The angiographic data will be examine at each study center by an independent radiologist or vascular surgeon with experience in interventional procedures. The primary safety endpoint is Freedom from device related Serious Adverse Events (SADEs) defined by the investigational site as part of normal reporting practices in any period between the procedure and 90 days after the procedure.

Secondary endpoints are listed in Table 2

**Table 2:** Secondary endpoints, ^1^PACCS, peripheral arterial calcium scoring system

Secondary endpoints
a. target vessel revascularization (TVR) at 3 and 12 months
b. target lesion revascularization (TLR) at 3 and 12 months
c. The change of the Rutherford classification [13] at 3 and 12 months compared to pre-procedure
d. freedom from any minor or major amputation at 3 and 12 months
e. therapy success of the recanalization of the iliaco-femoro-popliteal vascular flow path depending on the access (crossover/antegrade/retrograde)
f. evaluation of the recanalization result of superficial femoralis artery (SFA) stenosis/occlusions using IVUS vs. angiography for the purpose of deciding on therapy for additional Angioplasty/stent implementation/Drug Eluting Balloon (DEB)
g. the evaluation of the recanalization result of BYCROSS use without primary wire passage
h. the evaluation of the recanalization result of stenoses/occlusions in combination with hybrid interventions
i. the evaluation of the correlation between calcification (measeared and calcified in accordance with PACCS^1 ^[14] and procedural complications

### Technique

Technical aspects and application of the BYCROSS atherectomy system have been published previously [11, 12]. The BYCROSS™ has a coaxial, flexible, rotating shaft with an extendable tip and integrates an aspiration system for aspirating plaque debris and broken, as well as fallen thrombotic material. The expandable tip can increase the tip diameter from an outer diameter of 1.9 mm when closed to 4.7 mm when open. As the shaft rotates, the tip breaks the calcified atheroma or thrombus into small particles, which are simultaneously aspired into the guide sleeve and disposed of in the attached collection bag. Although BYCROSS is inserted via a guidewire, it does not require prior passage of an occlusion with the wire. Once the occluded segment is passed, the open-tip procedure is repeated on larger diameter vessels to further remove the remaining atheroma or thrombus. After passage of the lesion, regular angioplasty can be performed with or without a drug-eluting balloon.

### Adjuvant medical therapy

After the procedure, all patients receive aspirin 100 mg (1–0-0) and clopidogrel 75 mg (1–0-0) daily for at least 6 months. If there is something against this combination based on the patient-specific history (risk of falls, previous bleeding, use of oral anticoagulants such as Marcumar), individual adjustments will be made.

### Follow-up

All participants will be followed-up after successful intervention. Clinical assessments will be performed 90 days (± 14 days) and 12 months (± 2 weeks) after the procedure. The following assessments and/or procedures will be collected and reported for all subjects at follow-up visit.

- Stenosis assessment by Duplex Ultrasound
- Ankle-Brachial-Index (ABI) measurement, depending on the ABI a treadmill test or
- Transcutaneous oxygen partial pressure measurement (TcPO2)
- Rutherford classification [13]
- Target vessel revascularization (TVR)
- Target lesion revascularization (TLR)
- Measurement Quality of Live via EQ5D [15] questionnaire
- Measurement of pain via numeric pain scale (NRS) [16]
- Minor or Major Amputation needed
- Serious adverse events recording. Each event will be assessed whether root-cause for the event is related to device, procedure or not related.

### Safety

Serious adverse events (SAEs) occurring during the conduct of this study must be documented. The list of potential SAEs is provided in Table 3. All SAEs and serious adverse device events (SADEs) associated with the device will be recorded at each follow-up visit. Reporting will be in accordance with local requirements and policies. If the investigator identifies an SAE/SADE, an SAE reporting form must be completed and faxed or emailed to Taryag Medical (TARYAG-MEDICAL GmbH, Israel, malki@taryag-group.com) within 24 h of the investigator’s knowledge of the event.

**Table 3:** List of serious adverse events

Serious adverse events
a death
b serious deterioration in the health of the subject, that resulted in any of the following:
(I) life-threatening illness or injury,
(II) permanent impairment of a body structure or a body function,
(III) hospitalization or prolongation of patient hospitalization,
(IV) medical or surgical intervention to prevent life-threatening illness or injury or permanent impairment to a body structure or a body function,
(V) chronic disease
c Acute limb ischemia as a result of the procedure e.g. peripheral embolization
d Necessary thrombectomy/revision as a result of the procedure (surgical or interventional)
e Unplanned major amputation, above or below the knee

## Discussion

This study is designed as a multicenter, prospective, single arm, observational study to evaluate the success of the procedure and safety outcomes. A total of 60 (target 20 each center) individuals with a symptomatic PAD will be enrolled in the study.

The novel BYCROSS™ atherectomy represents a further development of existing atherectomy systems. During development, several limitations of other systems were identified and eliminated. The device does not require special guide wires and introducer sheaths, it is not necessary to first pass the occlusion with a guide wire. It operates without major equipment, pedal or nonsterile devices, and it is battery powered [17].

The safety and efficacy of the BYCROSS™ device was successfully tested in the Taryag Medical Clinical Investigation “BYCROSS™ Study” 2018/2019 (CIV-17–10-021851). Based on the BYCROSS trial and clinical experience, the BYCROSS device was shown to provide a solution with potentially minimal complications and improved treatment outcomes compared to simple balloon angioplasty (POBA) or stent-assisted POBA, especially in severely calcified vessels.

The procedural protocols of the clinical investigation demonstrated that the BYCROSS is a safe and effective option for intraluminal revascularization by avoiding vessel wall damage. Furthermore, no physical and mechanical changes of the BYCROSS were observed after the procedure at any PACCS [14] score or lesion length (up to 420 mm).

In the clinical investigation 42 patients with chronic limb ischemia and target vessel stenosis ≥ 80% with total or subtotal occlusion were enrolled and underwent procedure using the BYCROSS™ atherectomy device and adjunctive therapy, such as balloon and/or stenting if required.

Success of the acute intervention as the primary performance endpoint was assessed in each participating subject immediately after the intervention, and the primary safety endpoint was assessed after 30 days. Subjects were followed up over a 6-month period for assessment of the secondary safety endpoint and secondary performance endpoints. Thirty-nine of the 42 patients (in the full analysis (FA)) met the acute treatment success criterion. All patients were free of serious (major) device-related adverse events (MDAE) 30 days after the procedure. In conclusion, no serious device-related adverse events occurred in the clinical trial. The success rate of the acute intervention was over 92%.

## Conclusion

The purpose of this prospective national multicenter observational study is to collect further clinical data on the safety and performance of the BYCROSS atherectomy system and to monitor short- and mid-term treatment success.

The BYCROSS atherectomy system may be a novel device for the minimally invasive treatment of longstanding calcified lesions previously reserved for bypass surgery.

## Trial status

The trial has been registered in the German Clinical Trials Register (DRKS00029947), on 19 September 2022.The full WHO trial registration dataset is available via https://trialsearch.who.int/Trial2.aspx?TrialID=DRKS00029947. The protocol version is 2022–01; 20 May 2022, Recruitment began on 11 August 2022. The expected date for recruitment completion is December 2023.

## Data Availability

After publication of the study results, a fully anonymised data set and the statistical code can be made available upon justified scientific request and after ethical approval has been granted. Depending on the extent of the data use and the planned research, either appropriate credit or co-authorship must be granted to the authors of this study. A sample of the CRF used for this study can be provided upon justified scientific request.
